# Sequential Extraction of Valuable Bio-Products from Snow Crab (*Chionoecetes opilio*) Processing Discards Using Eco-Friendly Methods

**DOI:** 10.3390/md21060366

**Published:** 2023-06-20

**Authors:** Heather J. Burke, Francesca Kerton

**Affiliations:** 1 Centre for Aquaculture and Seafood Development, Fisheries and Marine Institute, Memorial University of Newfoundland, St. John’s, NL A1C 5R3, Canada; 2 Department of Chemistry, Memorial University of Newfoundland, St. John’s, NL A1C 5S7, Canada; fkerton@mun.ca

**Keywords:** snow crab, processing discards, eco-friendly, green extraction, vegetable oil, astaxanthin, chitin, carotenoprotein, marine bio-products, citric acid

## Abstract

Green extraction methods using a combination of mechanical, enzymatic, and green chemical treatments were evaluated for the sequential extraction of carotenoid pigments, protein, and chitin from crab processing discards. Key objectives included avoiding the use of hazardous chemical solvents, conducting as close to a 100% green extraction as possible, and developing simple processes to facilitate implementation into processing plants without the need for complicated and expensive equipment. Three crab bio-products were obtained: pigmented vegetable oil, pigmented protein powder, and chitin. Carotenoid extractions were performed using vegetable oils (corn, canola, and sunflower oils), giving between 24.85% and 37.93% astaxanthin recovery. Citric acid was used to demineralize the remaining material and afforded a pigmented protein powder. Three different proteases were used to deproteinate and isolate chitin in yields between 17.06% and 19.15%. The chitin was still highly colored and therefore decolorization was attempted using hydrogen peroxide. Characterization studies were conducted on each of the crab bio-products isolated including powder X-ray diffraction analysis on the chitin (80.18% crystallinity index, CI, achieved using green methods). Overall, three valuable bio-products could be obtained but further research is needed to obtain pigment-free chitin in an environmentally friendly manner.

## 1. Introduction

In Atlantic Canada, snow crab (*Chionoecetes opilio*) is the most important commercial species for the fishery and the rural economy, with an export value of ~CAD 1.3 billion in 2021 and annual landings of 76,828 tonnes [1]. Snow crab is primarily processed as Individually Quick Frozen (IQF) cooked sections, which generates 25–30% (~20,000 mt/year) waste comprised of carapace (cephalothorax shells), viscera and hepatopancreas, hemolymph, residual meat, and gills [2]. Crustacean processing discards contain valuable products including proteins, lipids, astaxanthin, organic acids, essential amino acids, chitin, and calcium [3]. In Atlantic Canada, snow crab processing discards are currently not being utilized commercially but could potentially be recovered from processing plant butchering stations as a by-product and converted into intermediate bio-products (crab meal, proteins, lipids, ash, chitin, pigments) or transformed into higher value bio-products (chitosan, peptides, omega-3, astaxanthin, marine calcium) (personal communication with industry stakeholders, [2,4]). These bio-products have a wide range of applications in several fields such as agriculture, aquaculture, biopharma, biomedicine, cosmetics, environment, food science and technology, and health and nutrition [2,5]. While most crab processing discards are being landfilled, or sea-dumped under permit, environmental restrictions are becoming more stringent, making it more difficult and costly for processors to continue this type of waste disposal (personnel communication with industry stakeholders, [4]). In addition, many of the processes used for crustacean valorization require hazardous chemical treatments using inorganic acids such as HCl, strong caustic solutions, and organic solvents such as hexane and ethanol [6,7,8,9,10], thereby raising other environmental concerns such as air and water pollution, and worker health and safety concerns [6,11].

Dedicated research efforts for the valorization of NL’s snow crab processing discards have not been published since 1991 [7] and these prior studies did not focus on green chemistry solutions. More recent studies on the valorization of Atlantic snow crab processing discards (2009–2011) have been conducted on crabs harvested from the North Atlantic between Greenland and Canada [9], and Gaspé, Quebec [12]. Most recently, a study was conducted by Nofima in Norway on snow crabs harvested from the Barents Sea, in 2021 [11]. In that study, a combination of extractions using edible oils and proteases were used to obtain astaxanthin and protein hydrolysates; however, traditional chemical solvents were used to extract chitin. In our approach we focused on green chemistry alternatives for all bio-products of interest, including chitin.

A key goal of this study was to identify opportunities to improve the utilization of crab processing discards generated by Newfoundland and Labrador (NL) processing plants, thereby keeping this material out of the environment and providing new economic opportunities for coastal communities. An additional goal was to consider inexpensive green technologies as an alternative to more harmful organic and inorganic chemical treatments that are traditionally employed for the recovery of crustacean bio-products. Replacing harmful chemicals with green alternatives is of particular importance to many NL coastal communities. A key issue is the transportation and storage of dangerous chemicals in areas where there are limited health and safety emergency response resources in the event of a chemical spill or chemical fire [personal communication with industry stakeholders, 4]. In addition to the environmental concerns of using harmful chemicals, there is an added health and safety concern for the intended end use of the bio-products due to possible contaminants from the chemical treatments used. Chemical treatments such as deproteination with NaOH and demineralization with HCl can also have a negative impact on the quality of the recovered bio-products by damaging the protein (e.g., hydrolysis and denaturation) and chitin molecules (e.g., decrease in molecular weight and degree of polymerization) [7,8].

This study focused on evaluating simple green technologies for the extraction of bulk intermediate bio-products from unseparated snow crab processing discards. Key objectives were to avoid the use of hazardous chemical solvents and to conduct as close to a 100% green extraction as possible for the valorization of snow crab processing discards. In this regard, inorganic hydrochloric acid was replaced with food-grade citric acid for shell demineralization, sodium hydroxide was replaced with food-grade protease enzymes for shell deproteination, and organic solvents (e.g., ethanol, hexane, acetone) were replaced with food-grade vegetable oils and biodegradable hydrogen peroxide for pigment recovery and chitin decolorization, respectively.

## 2. Results

### 2.1. Characterization of Raw Material (Crab Processing Discards)

The proximate composition and astaxanthin content of the unseparated crab discards are presented in Table 1. These results are comparable to results reported in the literature [7,9,10,13], except for total astaxanthin content. The astaxanthin content in the combined crab by-product as prepared in this study is lower than that reported elsewhere for specific crab shell components such as crab backs [7,9,10,13]. Therefore, separation of crab by-products at the processing plant may be required if pigment recovery is a priority.

### 2.2. Characterization of Extracted Crab Bio-Products

In this study, sequential extraction of carotenoid pigments, pigmented protein powder, and chitin from unseparated crab processing discards was attempted using green extraction methods. Direct pigment extraction from crab shell waste was carried out using various refined vegetable oils prior to protein and chitin extraction to avoid degradation of the carotenoid pigments. A similar approach was used by Shahidi and Synowiecki [7] using cod liver oil to extract carotenoids from shrimp (*Pandalus borealis*) shell waste.

#### 2.2.1. Extraction of Carotenoids with Vegetable Oil

Three common vegetable oils were selected based on availability from Canadian producers and wholesale price (in USD), namely canola oil (0.77 USD/kg), corn oil (0.67 USD/kg), and sunflower oil (0.54 USD/kg) (https://www.selinawamucii.com/, accessed on 19 February 2022). Fish oil was also considered but was not included due to its higher cost (2.92 USD/kg) (tridge.com, accessed on 19 February 2022) and limited availability.

The recovery of astaxanthin from crab processing discards using different vegetable oils is presented in Table 2. Recovery is reported as the percent of total astaxanthin in the original crab by-product sample (21.094 μg/g wet basis). The highest astaxanthin recovery was obtained using corn oil (37.93% ± 0.04) followed by canola oil (31.23% ± 0.03) and sunflower oil (24.85% ± 0.01). These values are lower than those reported in the literature where the recovery of carotenoids from shell waste using vegetable oils and marine oils ranged from 40 to 74% [7,14,15,16].

Based on the astaxanthin recovery obtained in this study, corn oil and canola oil performed better than sunflower oil. The results show that corn oil was most effective for recovering astaxanthin from crab processing by-products using a single-stage extraction in a 1:1 (*v*/*w*) ratio of oil to waste, at 60 °C for 2 h. Sunflower oil did not perform as well as reported elsewhere for shrimp [16,17] and was the least effective in recovering astaxanthin from the crab by-product material under the study extraction parameters. An interesting observation was the odor of the pigmented oils. All pigmented oils had strong crab meat and fishy aromas. According to Cheng and Zhang [18], the two major odorants in Chinese mitten crab with high odor intensity were dimethyl sulfide (crab meat aroma) and TMA (fish and amines odor). These highly odiferous pigmented oils may therefore be useful as an attractant in aquaculture feeds, or as a flavoring in soup bases or formulated crab-based foods [5]. If the intent, however, is for use in a nutraceutical product, or as a colorant in beverages, the oil would require a deodorization step.

#### 2.2.2. Pigmented Protein Powder

The composition of the pigmented protein powders recovered following carotenoid extraction with vegetable oils and demineralization with citric acid is provided in Table 3. The protein contents of all three samples were ~51% (db) and were not statistically different (F_2,3_ = 0.64, *p* = 0.59). The low ash contents (<1%) indicate that demineralization was effective for removal of minerals. The astaxanthin content varied between the samples and was significantly different (F_2,6_ = 48.60, *p* < 0.0002) for all three samples. About 56–66% (db) of the total astaxanthin was retained in the protein fraction. The astaxanthin content of the pigmented protein powders was in the order of sunflower > canola > corn, confirming that corn oil was most effective for the recovery of astaxanthin from crab processing by-products, followed by canola oil and sunflower oil.

Our results show that the vegetable oil extraction method was not efficient for full recovery of astaxanthin, which may be the result of its association with protein in the form of a water-soluble pigment–protein complex, or carotenoprotein, in which the pigment and protein are associated via ionic bonding [19,20]. This carotenoprotein complex is thought to stabilize carotenoids [21,22,23], making them less susceptible to photo-oxidation [19,24]. Organic solvents such as ethanol and acetone can split the carotenoprotein into carotenoid and apoprotein, facilitating extraction of the lipophilic astaxanthin molecule [15,19,25]. Shahidi and Synowiecki [7] reported that astaxanthin is found in both the free (21.16%) and esterified forms (61.68%) in crab by-products, with astaxanthin diester (56.57%) being the major carotenoid present. However, since astaxanthin mainly exists as a carotenoprotein in crustacean shells [16,26], a longer heat treatment and/or higher temperature may be required to disrupt the ionic bond and optimize astaxanthin extraction using vegetable oil solvents.

The high protein (~51%), lipid (~16–25%), and astaxanthin contents (33.8–39.6 µg/g) plus the low ash content (<1%) of the protein-pigment powders may make them suitable for use in aquaculture feeds and poultry feeds. By calculation, the pigmented protein powders contain ~22–32% (db) carbohydrate, likely chitin, which could affect the nutritional properties and applications as an ingredient. Characterization of the amino acid composition, fatty acid composition, lipid profile, and composition of the carotenoid pigments will be critical in making this determination.

#### 2.2.3. Chitin

Characteristics of chitins prepared from crab processing discards using green extraction methods are presented in Table 4. In the current study, the oil treatment did not affect the chitin yield (F_2,6_ = 1.097, *p* = 0.39). However, the enzyme treatment did have a significant effect (F_2,6_ = 7.73, *p* = 0.022) on chitin yields, which were 1.5–2% higher for samples treated with Weifang protease enzyme in comparison to FAP (Fungal Acid Protease) and Sea-B-Zyme. For comparison, chitin samples were also extracted from snow crab processing discards using the normal chemical method [6,7], which included demineralization with HCl, deproteination with NaOH, and depigmentation with acetone. Characteristics of chitins prepared using the chemical method are presented in Table 5. Chitin yields using the green extraction method ranged from 17 to 20% (db) (Table 4) and were comparable to chitin yields obtained using a traditional chemical extraction method (16–18% db, Table 5).

##### Ash Content

The demineralization reaction between calcium carbonate and citric acid produces calcium citrate, carbon dioxide, and water (Equation (1)). The amount of acid must be stoichiometrically equal to or greater than all the minerals present in the sample to ensure complete reaction, which depends on the acid concentration and the ratio of shell to acid [6,7]. For stoichiometric calculations, it was assumed that all mineral deposits were due to calcium carbonate. In this study, 1.5× the stochiometric amount of citric acid needed for demineralization was attempted and applied in a two-step process.

3CaCO_3 (s)_ + 2H_3_C_6_H_5_O_7 (aq)_ ↔ Ca_3_(C_6_H_5_O_7_)_2 (aq)_ + 3H_2_O _(l)_ + 3CO_2 (g)_ (1)

The two-step citric acid process used in this study effectively removed 94–98% of the mineral content from the crab shells; however, full demineralization was not achieved. This could be due to the presence of insoluble calcium citrate formed during step one of the reaction sequences (pH of 3.06–3.50). A residual ash content below 2.5% is required for food-grade chitin [6], and a lower target of <1% residual ash is recommended for higher quality applications such as chitosan [6]. The ash contents of the green extracted chitin samples ranged between 0.75% (db) and 2.16% (db), meeting food-grade quality; however, only one sample (Canola + FAP) met the residual ash content recommended for chitosan applications. The ash contents of the chemically extracted chitin samples were all below 1% and ranged from 0.26% to 0.68% (db) (Table 5).

##### Total Nitrogen Content

The total nitrogen content of the “green extracted” chitin samples ranged from 6.19 to 6.61% (db) which is comparable to that reported by Shahidi and Synowiecki at 6.42–6.48% (db) [7], and similar to the results obtained for our chemically extracted chitin samples (6.30–6.43% db, Table 5). The effect of the oil treatment was not significant (F_2,9_ = 0.17, *p* = 0.85), and there was no significant interaction (F_2,9_ = 2.38, *p*= 0.13) between the oil treatment and enzyme treatment on total nitrogen content. However, the enzyme treatment did have a significant effect (F_4,9_ = 8.84, *p* = 0.0075) on total nitrogen content of the samples, with the Weifang-treated samples having higher total nitrogen contents overall.

##### Residual Protein Content

Residual protein content ranged from 0.26 to 0.95% (db); however, these differences were not significant (F_4,27_ = 0.28, *p* = 0.89). Residual protein content was highest in Weifang- (0.63–0.95% db) treated samples, followed by Sea-B-Zyme- (0.39–0.77% db) and FAP- (0.26–0.45% db) treated samples, respectively. Shahidi and Synowiecki [7] reported 0.45–0.49% (db) protein residue in crab shell chitin obtained using a traditional chemical process. We obtained residual protein contents of 0.12–0.35% (db) for our chemically extracted crab chitin samples. The results of the current study indicate that treatment with various protease enzymes is effective for reducing the residual protein in crab chitin samples to <1% and can be used as a replacement for chemical deproteination with NaOH. The selection of the enzyme will therefore depend on the cost of the enzyme treatment and the targeted chitin application. If a lower residual protein content is required, it may be possible to optimize the enzyme treatment by adjusting the enzyme concentration and time–temperature application. Based on these results, Weifang acid protease is recommended for industrial chitin applications. Sea-B-Zyme and FAP can be considered for biomedical chitin applications. Another approach used in other studies is to treat the chitin with dilute NaOH solutions to remove any residual protein following enzymatic deproteination [11]. However, the goal here is to use as close to 100% green chemistry as possible, thus avoiding the use of corrosive extraction chemicals.

##### Chitin Nitrogen Content

The nitrogen content of pure chitin is 6.9%. The chitin nitrogen contents of the green extracted chitin samples in this study were lower, ranging from 6.07 to 6.46% (db), but are comparable to the chitin nitrogen content obtained using chemical methods (6.23–6.43% db). This is a good result but indicates that the chitin samples contain impurities (e.g., ash, protein), and that our green extraction process requires additional optimization. The enzyme treatment had a significant effect on chitin nitrogen (F_2,15_ = 3.75, *p* = 0.048), with FAP- and Weifang-treated chitins having similar and higher chitin nitrogen contents in comparison to Sea-B-Zyme-treated chitins. The Corn Oil-Sea-B-Zyme-treated chitin had the lowest chitin nitrogen content, while the highest was obtained for the Corn Oil-Weifang-treated sample. The oil treatment had no effect on chitin nitrogen (F_2,15_ = 3.12, *p* = 0.07).

##### Color Characteristics

**Figure 1:**
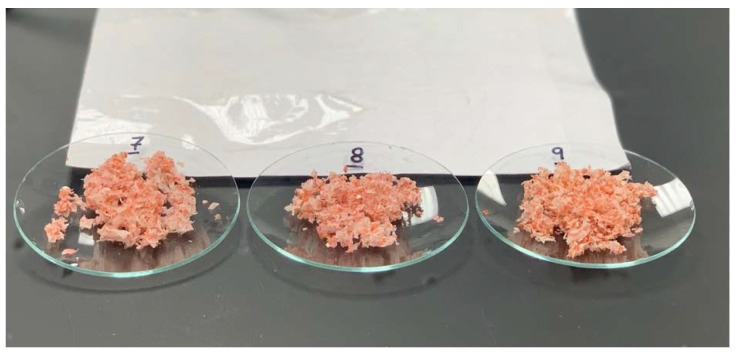
Chitin samples treated with 37% hydrogen peroxide.

**Figure 2:**
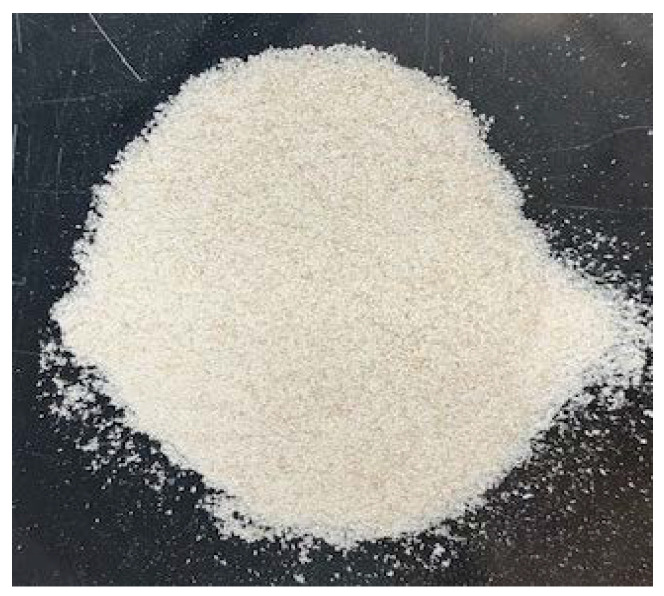
Chemically extracted crab chitin.

The color of the final chitin samples was a mix of off-white, light pink, and pink (Figure 1), indicating that decolorization with hydrogen peroxide was not effective. The desired characteristic white to off-white color (Figure 2) was not achieved using the green extraction methodology employed. Evaluation of the total astaxanthin content confirmed that the chitin samples contained 9.91–20.70 µg/g (db) total astaxanthin, representing 16.5–34.5% (db) retention of the total astaxanthin from the original crab by-product. Weifang-treated chitin samples had the lowest astaxanthin contents (9.91–13.12 µg/g) overall.

Two-factor ANOVA indicated that both the oil treatment (F_2,18_ = 15.57, *p* = 0.0001) and the enzyme treatment (F_2,18_ = 1187.59, *p* < 0.0001) had a significant effect on the astaxanthin content of the extracted chitin, and that there were significant crossed effects between the oil and enzyme treatments (F_4,18_ = 8.44, *p* < 0.0001). The Sunflower Oil-Weifang-treated chitin sample had the lowest astaxanthin content (9.91 µg/g) of all the samples. The effect of protease enzymes on pigment removal is likely due to hydrolysis of carotenoproteins.

The Hunter color characteristics of the prepared chitin samples were also evaluated. Two-factor ANOVA indicated that both the oil and enzyme treatments had a significant effect (*p* < 0.05) on the color characteristics of the chitin, and the interaction between the oil and enzyme treatment was also significant (*p* < 0.05). Further ANOVA analysis identified the following: (1) the oil treatment had a significant effect (F_2,51_ = 3.81, *p* = 0.03) on the L-values with the corn oil treated samples having higher L-values overall, indicating these samples are lighter (whiter) in color; (2) the Weifang-treated samples had different a-values (F_2,51_ = 26.01, *p* < 0.0001) than FAP- and Sea B Zyme-treated samples, with a-values being slightly lower overall, indicating the Weifang samples are less red; and (3) b-values were significantly different (F_2,51_ = 12.15, *p* < 0.0001) for the sunflower-oil-treated samples which were higher overall all, indicating these samples are more yellow.

A regression analysis (Table A1, Appendix A) was conducted to determine if there is a correlation between the astaxanthin content and the L*a*b values of the chitin samples. The results indicate that astaxanthin and L-values (whiteness) are not correlated (r = −0.19), and astaxanthin and b-values (yellow-blue) are not correlated (r = −0.29). However, astaxanthin and a-values (redness) have a moderate positive correlation (r = 0.598), L-values and a-values have a moderate negative correlation (r = −0.64), and a-values and b-values are slightly positively correlated (r = 0.48). Therefore, a-values (redness) increase with increasing astaxanthin content and decrease with increasing L-values (whiteness). Astaxanthin content, however, and L-values (whiteness) are independent of each other. This suggests that whiteness of the chitin samples is affected by the hydrogen peroxide treatment, whereas redness is a result of the astaxanthin in the sample, which is affected by the oil and enzyme treatments.

##### X-ray Diffraction Pattern and Crystallinity Index

X-ray diffraction (XRD) has been used to characterize the crystalline structure of chitin [27,28,29]. For the current study, XRD patterns and crystallinity index (CI) for selected crab shell chitins extracted using a traditional chemical method were compared with green extracted crab shell chitins (Table 4). The XRD patterns were characteristic of α-chitin with reflections at 9–10°, 19–21°, and ≥26° [30]. The crystallinity index (% CI) values of the extracted chitins were not statistically different (F_1,4_ = 1.10, *p* = 0.35) and ranged from 80.18 to 93.18% CI (Table 6). The crystallinity index of all the samples was >80% (Table 5), which is typical for α-chitin [31]. The green extracted chitin sample had the lowest % CI (80.18%) and the highest amorphous diffraction intensity at 2θ = 12.6° (611.11), suggesting partial degradation of the chitin molecule, presumably due to hydrogen peroxide treatment. H_2_O_2_ has been shown to decrease the crystallinity of chitosan due to structural rearrangements, decrease MW, decrease the degree of polymerization, and increase solubility [32].

## 3. Discussion

In this study, sequential extraction of carotenoid pigments, pigmented protein powder, and chitin from unseparated crab processing by-products was attempted to facilitate ease of raw material collection, which is an important consideration for industry. Results of the protein, ash, lipid, and chitin content of our snow crab processing discards (Table 1) are comparable to those reported in other research studies [7,9,10,13]. However, the astaxanthin content of our unseparated samples is much lower than that reported for separated crab by-products such as crab shell backs [7,9,10,13]. This suggests that separation of crab by-products at the processing plant may be required, particularly if pigment recovery is a priority.

Traditional technologies using organic and inorganic solvents as reported elsewhere for the extraction of crustacean bio-products such as astaxanthin can be expensive and inflexible and may cause structural changes in valuable compounds resulting in a loss in functionality or a decrease in nutritional value [17,33,34]. A promising alternative for the extraction of astaxanthin is the use of edible oils since astaxanthin is oil-soluble [15,17]. Edible oils may also protect the pigment against oxidation, and act as a pigment carrier and an energy source in aquaculture feed [15,35]. However, previous studies using vegetable oil alternatives reported lower yields of carotenoids from crustacean processing wastes than those obtained using organic solvents [7,14,15,16,17]. Parjikolaei et al. [17] have suggested that the lower yields are due to the high viscosity of vegetable oils resulting in less diffusivity and point to a lack of comprehensive studies on effective extraction methods and optimized processing conditions using vegetable oil solvents.

To avoid degradation of the carotenoid pigments, we decided to perform direct pigment extraction, using vegetable oils, from crab processing discards prior to protein and chitin extraction. Using a single stage extraction process with a 1:1 (*w*/*v*) of discards:oil and heating at 60 °C for 2 h we obtained 24.85%, 31.23%, and 37.93% astaxanthin recovery with sunflower oil, canola oil, and corn oil, respectively (Table 2). Shahidi and Synowiecki [7] reported that the best recovery of carotenoids (74.23%) from shrimp shell waste was obtained using a ratio of 1:2 (*w*/*v*) offal:fish oil at 60 °C. Chen and Myers [14] reported ~40–52% carotenoid recovery from crawfish shell wastes using a single-stage extraction process with soybean oil in a 1:1 (*v*/*w*) ratio of oil:shell waste and heated at 80–90 °C for 30 min [14]. Sachindra and Mahendrakar [15] evaluated several vegetable oils for the extraction of carotenoids from shrimp shell wastes and obtained higher carotenoid yields using refined sunflower oil in an oil to waste ratio of 2:1 and heating the mixture at 70 °C for 150 min. Hooshmand et al. [16] also reported higher yields of carotenoids from crab wastes using a multi-stage extraction process with sunflower oil in a ratio of 5:1 (*v*/*w*) oil to waste at 78 °C for 95 min in comparison to other vegetable oils, but extraction using organic solvents such as acetone was more efficient than vegetable oil extraction. In addition, Hooshmand et al. [16] reported carotenoid yields were higher in shrimp wastes than in crab wastes. In our study, the only variable evaluated was the type of vegetable oil used for extraction. However, many factors can affect optimization of pigment extraction (e.g., time, temperature, solvent viscosity, particle size). Further evaluations using corn and canola oils as solvents for pigment extraction and recovery are recommended and should focus on (1) optimizing the crab:oil ratio; (2) comparing single- vs. multi-stage extraction processes; (3) determining the effects of moisture, particle size, time, and temperature on pigment recovery; and (4) determining the effect of using co-solvent mixtures such as vegetable oil and ethanol to reduce viscosity. Soybean oil was not available for this study but may be worth further evaluation due to the earlier success noted by Chen and Meyers [14] for extraction of astaxanthin from crawfish shell waste.

The high protein (~51%), lipid (~16–25%), and astaxanthin contents (33.8–39.6 µg/g) plus the low ash content (<1%) of the protein-pigment powders may make them suitable for use in aquaculture feeds and poultry feeds. However, further characterization of the protein powders is needed, including chitin content, amino acid, and fatty acid profiles, and heavy metals, to confirm nutritional quality. In a previous study [4] we determined that crab meal powders were low in two essential amino acids, methionine and lysine. Therefore, the crab protein powder will likely be low in these amino acids. We also established that crab meal and enzymatically obtained protein hydrolysates were high in arsenic, exceeding regulatory limits for use in feeds, foods, and natural health products [36]. Therefore, heavy metal testing will be a key quality parameter requiring further evaluation. It is not known if the high lipid contents of the protein powders are the result of the vegetable oil treatment or the result of crab oil naturally present in the raw material. The lipid contents in the powders represent 26.68–42.94% (*w*/*w*) of the original lipid content in the crab by-products with the corn-oil-treated sample having the lowest percentage. Further characterization of the pigmented protein powders will be a key factor in further identifying opportunities for commercial applications.

The two-step citric acid process used in this study effectively removed 94–98% of the mineral content from the crab shells; however, full demineralization was not achieved. A slightly higher concentration of acid, or lower pH (pH < 3), and a higher ratio of acid to shells will be required to reduce the ash content of snow crab shells to <1%. This target was initially achieved in an earlier study [4] using a two-step demineralization process with 7.5% (*w*/*v*) citric acid and 1:10 ratio of shells to acid (4.5x stochiometric amount of acid). However, double demineralization using 5% (*w*/*v*) citric acid added in a 1:5 ratio of shells to acid (1.5x stochiometric amount of acid) was less effective, leaving some residual mineral content in the resulting chitin. These preliminary results are a good indication that citric acid can effectively replace HCl as a green solvent for crab shell demineralization. A similar result was reported by Pohling et al. [6] using a two-step citric acid demineralization process for shrimp shells (*Pandalus borealis*). Further research to optimize a citric acid two-step demineralization process for snow crab by-products is therefore recommended. For example, Hajiali et al. [37] successfully demonstrated that citric acid used in a mechanochemistry process can be used to obtain chitin with high yields and low ash content from European green crab, which may be applicable to snow crab.

Comparison of the green extracted chitin with chemically extracted chitin indicates that the % chitin (16–20% db), % total nitrogen (6.19–6.55% db), and % chitin nitrogen (6.07–6.46% db) are similar regardless of extraction method used. The residual protein content was <1% in all chitin samples, but was slightly higher in the protease-treated chitin samples vs the NaOH-treated chitin samples. With further optimization of the enzymatic deproteination step, a lower residual protein content may be obtained. Therefore, sequential chitin extraction using vegetable oil and protease enzymes can be just as effective as the chemical approach, with the added benefit of being eco-friendly.

All three processing treatments (oil, enzyme, and hydrogen peroxide) influenced the color characteristics of the chitin samples. The oil treatment had a significant effect on the L-values and the b-values, whereas the enzyme treatment had a significant effect on the a-values. This suggests that the combined effects of the oil treatment and the enzyme treatment could be further optimized to recover more astaxanthin in the pigmented oils and facilitate further pigment removal during deproteination. The combined effect of corn oil treatment with Weifang enzyme had the most significant effect on the color characteristics of the chitin in terms of whiteness and redness, thus justifying further investigation. It may be possible to eliminate a final decolorization step if the oil and enzyme treatments can be optimized to maximize pigment recovery (oil treatment) and subsequent removal of residual color (enzyme treatment). The results also suggest that H_2_O_2_ may be more effective as a whitening agent when more astaxanthin is removed from the samples prior to H_2_O_2_ treatment. The H_2_O_2_ treatment may have some effect on color removal based on the correlation between L-values and a-values; however, peroxide treatment alone was not effective for full decolorization of the chitin samples. It may be possible to optimize treatment with hydrogen peroxide considering the effects of other process variables such as particle size, shell to liquid ratio, time, and temperature, in addition to oil and enzyme treatments.

X-ray diffraction (XRD) has been used to characterize the crystalline structure of chitin, which varies depending on the source, with α-chitin being the most abundant form in nature, and the predominant form found in shrimp and crab shells [27,28,29]. Characterized by strong intermolecular hydrogen bonding and a structure of antiparallel chains in the crystalline regions, α-chitin is highly crystalline and unable to swell in water, limiting its bioactivity [27,32,38]. Jang et al. [39] studied the XRD pattern of α-chitin and identified four sharp crystalline reflections at 9.6, 19.6, 21.1, and 23.7°. Abdou et al. [30] reported strong reflections around 9–10° and 20–21°, and minor reflections at higher 2θ values of ≥26.4°. XRD patterns and the crystallinity index of our chitin samples were characteristic of α-chitin [30,31]. However, our “green” extracted chitin sample had the lowest % CI (80.18%) and the highest amorphous diffraction intensity at 2θ = 12.6° (611.11), suggesting partial degradation of the chitin molecule. H_2_O_2_ has been shown to decrease the crystallinity of chitosan due to structural rearrangements, decrease MW, decrease the degree of polymerization, and increase solubility [32]. Future studies should focus on optimizing the enzyme–oil–citric acid combination to try and obtain a less red chitin product and minimize the amount of H_2_O_2_ or eliminate it, to make the process even more environmentally safe.

## 4. Materials and Methods

Crab processing discards were collected in May 2021 from a processing plant in Bay de Verde, Newfoundland, Canada. Following preparation and stabilization of the crab discards, this raw material was characterized as described in Section 4.1. A combination of mechanical, enzymatic, and green chemical treatments was used to extract carotenoids, proteins, and chitin as described in Section 4.2.

### 4.1. Characterization of Raw Material

#### 4.1.1. Proximate Composition of Raw Material

Proximate composition of the crab by-product raw materials was determined using the following AOAC methods: Moisture Content-AOAC Method 930.14; Kjeldahl Nitrogen-AOAC Method 954.01/988.05; Lipid Content-AOAC 920.39 (Soxhlet); and Ash Content-AOAC Method 938.08 Ash of Seafood.

#### 4.1.2. Chitin Content

Chitin content was determined following demineralization of 5–10 g of crab by-product with 50–100 mL of 7% HCl for 3 h at 25 °C, followed by deproteination with 10% NaOH (1:8 of crab:NaOH) for 2–3 h at 55–60 °C [6,7]. The chitin was collected on Whatman No. 4 filter paper using a Buchner funnel and washed a minimum of three times with deionized water to pH 7, followed by oven drying at 105 °C for 24–48 h.

#### 4.1.3. Characterization of Chitin

The recovered chitin was analyzed for total nitrogen via the Kjeldahl method (AOAC 954.01/988.05), residual protein nitrogen via the Lowry method, and ash content (AOAC 938.08). Chitin nitrogen was calculated using Equations (2) and (3).

% Chitin Nitrogen = % Total (Kjeldahl) Nitrogen − % Protein Nitrogen(2)

% Protein Nitrogen = % Residual (Lowry) Protein ÷ 4.94(3)

where 4.94 is the nitrogen-to-net protein conversion factor for fish and fish products [40].

#### 4.1.4. Total Astaxanthin Content

Astaxanthin was extracted from the raw crab by-product samples using hexane:isoproponal (3:2 *v*/*v*) as described by Sindhu and Sherief [41]. Immediately following extraction, the samples were placed in a cuvette and the absorption was measured at λ_max_ (476 nm) in a HACH DR600 Spectrophotometer. The pigment concentration was calculated using Equation (4) and reported as total astaxanthin.

(4) $$Astaxanthin\left[\frac{\mu g}{g}\right]=\frac{A*D*10^{6}}{100*G*d*E}$$

where A = absorption at λ_max_, D = volume of extract [ml], G = sample weight [g], d = cuvette distance (10 mm), E = extinction coefficient [41].

### 4.2. Extraction of Crab Bio-Products

Extraction of crab bio-products involved simple processes using a combination of mechanical, enzymatic, and green chemical treatments to recover carotenoids (astaxanthin), proteins, and chitin as described in Section 4.2.1, Section 4.2.2, Section 4.2.3 and Section 4.2.4.

#### 4.2.1. Astaxanthin Extraction in Vegetable Oil

Astaxanthin, the principal carotenoid in crustaceans, is a lipid soluble orange-red pigment found in both the free and esterified forms in crustacean shells [7,26]. Its solubility in fats and oils is due to its long unsaturated aliphatic chains. Because of this lipophilic property, different vegetable oils were selected as green solvent alternatives to replace the use of flammable solvents such as ethanol and acetone, which are often used for the extraction of natural health products and for depigmentation of chitin.

An amount of ~100 g of crab by-product was blended with 50 mL distilled water in a Ninja blender to a particle size of 1–5 mm. The blended crab samples were mixed with each oil in a 1:1 (*w*/*v*) ratio of crab by-product:oil in 500 mL glass Mason jars and incubated for 2 h in a Thermo Scientific (Waltham, MA, USA) MaxQ 6000 Shaker at 60 °C with continuous agitation at 165 rpm [7,14,15]. After incubation, each sample was transferred to a 500 mL centrifuge bottle and centrifuged in a Thermo Scientific Sorvall Lynx 4000 Centrifuge for 10 min at 8000 rpm and 20 °C. The pigmented oil layer was carefully decanted, and gravity filtered through Whatman #40 ashless filter paper and collected in a 125 mL Erlenmeyer flask. The volume of pigmented oil recovered was noted. The aqueous layer was discarded. The pigmented oils were collected in 50 mL centrifuge tubes, wrapped in foil, and stored at −80 °C until required for testing.

The astaxanthin content in the pigmented oils was measured spectrophotometrically at 470 nm against the oil used as a blank [15]. The total astaxanthin content was calculated using Equation (4).

#### 4.2.2. Demineralization with Citric Acid

Following astaxanthin extraction, the remaining solids were collected on a 45 mesh (355 µm) screen and washed several times with distilled water to remove residual oil. The collected solids contained protein, shell, and some residual oil. This material was demineralized using citric acid (H_3_C_6_H_5_O_7_—an organic acid found in citrus fruit) instead of hydrochloric acid (HCl—a strong inorganic acid traditionally used for shell demineralization) in a two-step demineralization process. The pH of mixtures was monitored throughout using a HQ40D portable multi-meter (HACH, London, ON, Canada).

Crab shells were mixed with aqueous citric acid (5% *w*/*v*) in a 1:5 ratio of crab:solution at room temperature for 30 min. The citric acid was added in 2 equal portions to avoid excess foaming. The mixture was stirred at room temperature using a magnetic stirrer for ~30 min and until foaming had stopped. After 30 min, the shells were drained, rinsed, and mixed with fresh aqueous citric acid (5% *w*/*v*) and allowed to react at room temperature until there was no change in pH (~90 min). The samples were then drained, washed, and pressed through a 1 mm and 0.5 mm sieve to collect the demineralized shell (>1 mm) and the residual protein fraction (<1 mm). Each fraction was washed several times with distilled water to pH 7 and pressed to remove excess moisture. The protein fraction was dried in a convection oven at 55 °C overnight to constant weight.

#### 4.2.3. Enzymatic Deproteination

Following demineralization, the recovered shell material was enzymatically deproteinated using three different proteases, replacing the traditional method of deproteinating with KOH or NaOH [7]. The enzymes selected for this study were Fungal Acid Protease (FAP) *Aspergillus oryzae* purchased from Sigma-Aldrich, Burlington, MA, USA, Sea-B-Zyme L200 purchased from Specialty Enzymes (Chino, CA, USA), and Acid Protease from Weifang Yuexiang Chemical Co. Ltd., Weifang, China and provided by Ensymm UG & Co. KG, Düsseldorf, Germany.

The demineralized shells were divided into 3 equal portions for treatment with each of the 3 selected protease enzymes. The enzyme characteristics and reaction parameters for each enzyme treatment are presented in Table 7.

The pH of the demineralized shells was monitored using a HQ40D portable multi-meter (HACH, London, ON, Canada). The demineralized shells were added to a 500 mL glass Mason jar and either mixed with distilled water or phosphate buffer depending on pH, in a ratio of 1:10 shells:liquid *w*/*v*. The pH was monitored and adjusted as needed according to the parameters outlined in Table 1. The shell:liquid mixture was heated to 45 °C, at which time the enzyme was added. The jars were capped and incubated for 2 h in a Thermo Scientific Enviro Shaker at 45 °C with continuous agitation at 165 rpm. The pH was monitored at 30 min intervals and adjusted using 1M HCl or 1M NaOH as required. Enzyme deactivation was achieved by heating the mixture to 80 °C and holding for 20–30 min. The samples were removed from the incubator and cooled to room temperature.

After enzyme deactivation and cooling, the shells (crude chitin) and filtrate (protein hydrolysate) were separated by vacuum filtration on Whatman #40 ashless filter paper. The filtrate (protein hydrolysate) was collected in 50 mL centrifuge tubes, capped, wrapped in foil, and stored at –80 °C. This represents a protein hydrolysate product which can be spray dried or freeze dried into a powder containing 50–60% protein and 20–25% mineral [36]. The chitin was washed several times with distilled water to pH 7 and vacuum filtered.

#### 4.2.4. Decolorization with Hydrogen Peroxide

Decolorization of the wet chitin fraction was attempted using laboratory grade 27% H_2_O_2_. Hydrogen peroxide was selected as an alternative to flammable solvents (e.g., acetone, ethanol, hexane) and hazardous bleaching agents (e.g., NaOCl) that are traditionally used for chitin depigmentation and decolorization [7]. Hydrogen peroxide is a strong bleaching agent that breaks down into water and oxygen leaving no harmful by-products and it is biodegradable, therefore meeting our selection criteria for green reagents and conditions. Chitin samples were placed in 500 mL Mason jars, mixed with 27% H_2_O_2_ in a ratio of 1:4 chitin:H_2_O_2_, and heated to 30 °C using a VWR hot plate stirrer. The jars were capped and incubated for 2 h in a Thermo Scientific Enviro Shaker at 30 °C with continuous agitation at 165 rpm. The samples were removed from the incubator, cooled to room temperature, vacuum filtered using Whatman #40 ashless filter paper, washed with distilled water to a final pH of 7, transferred to a watch glass, and dried at 55 °C overnight to a constant weight.

### 4.3. Characterization of Snow Crab Bio-Products

Selected snow crab bio-products were characterized using the methods outlined in Table 8 and further described in Section 4.3.1, Section 4.3.2 and Section 4.3.3.

#### 4.3.1. Tristimulus Color Parameters

The tristimulus color parameters, L (lightness/darkness), a* (red /green), and b* (yellow/blue) of ground chitin and protein-pigment powder samples were measured using a portable handheld ColorTech-PCM Colorimeter (ColorTec, Clinton, NJ, USA), with a measurement angle of 10°, Illuminator D65, and aperture of 8 mm. The chromatic properties were defined by the L*a*b* color method of the CIE (Commission Internationale de l’Eclairage) and were expressed as L* (lightness; 100 = white, 0 = black), a* (red +; green -), and b* (yellow +; blue -) coordinates. Each sample was milled to a particle size of ~0.5 mm using an IKA WERKE MF 10 basic Microfine grinder equipped with a MF 1.2 impact grinding head and MF 0.5 stainless steel sieve. Approximately 2 g of each milled sample was placed on a watch glass. The dishes were placed on a white surface and measurements were carried out in at least triplicate.

#### 4.3.2. Powder X-ray Diffraction

Powder X-ray diffraction was used to compare the effect of chemical and green extraction methods on the crystalline structure of the extracted chitin. X-ray powder diffractograms for chitin were recorded with a Rigaku Ultima IV automated X-ray diffractometer with a copper X-ray source (40 kV/44 mA current) and a scintillation counter detector. The diffraction profile was recorded at room temperature at a scan speed of 1.0 deg/min, scan axis of 2theta, and scan range of 3–100°. The crystallinity index (CI) was calculated using Equation (5) where I_110_ is the maximum intensity of the (110) peak at around 2theta = 19°, and I_am_ is the amorphous diffraction at 2theta = 12.6° [27,42,43].

CI (%) = [(I_110_ − I_am_)/I_110_] × 100 (5)

#### 4.3.3. Chitin Yield

Chitin yield of the recovered chitin was calculated for crab meal using Equation (6).

% Chitin Yield = [weight of chitin (g)/weight of crab by-product (g)] × 100(6)

### 4.4. Statistical Analysis

Results were compared using analysis of variance (ANOVA) and the differences between the means by Tukey’s test. All analyses were performed using the Data Analysis ToolPak in Microsoft Excel for Mac, Version 16.44. Alpha level 0.05 was selected as the threshold of significance to test the null hypothesis that all sample means are the same.

## 5. Conclusions

Edible oils, food-grade citric acid, and food-grade proteases are promising alternatives for the green extraction of carotenoid pigments, pigmented protein powder, and chitin from snow crab processing discards. These green chemical treatments have potential to replace harmful organic solvents (e.g., acetone, ethanol) and inorganic reagents (e.g., HCl, NaOH) traditionally used for extracting crustacean bio-products. Results of our crab bio-product characterization studies demonstrate that with further optimization and scale-up studies, green extraction methods can offer effective, eco-friendly, and safer alternatives to traditional chemically intensive approaches for valorizing snow crab processing discards. However, challenges remain in terms of developing environmentally friendly chitin decolorizing technologies and this area needs further investigation.

## Figures and Tables

**Table 1:** Composition of crab processing discards collected in May 2018 and May 2021.

Parameter	May 2018 ^5^	May 2021 #1 ^5^	May 2021 #2 ^5^	*F* Value	*p* Value
%Moisture Original Sample ^1^	65.60 ± 0.41 ^a^	65.23 ± 0.85 ^a^	65.12 ± 0.34 ^a^	F_2,6_ = 4.36	0.07
% Ash (db) ^2^	34.40 ± 0.45 ^a^	37.06 ± 1.10 ^b^	38.06 ± 1.60 ^b^	F_2,6_ = 8.13	0.019
% Nitrogen (db) ^2^	5.93 ± 0.08 ^a^	5.30 ± 0.16 ^b^	5.40 ± 0.23 ^b^	F_2,6_ = 12.60	0.007
% Lipid (db) ^2^	3.22 ± 0.63 ^a^	2.04 ± 0.002 ^a^	2.19 ± 0.23 ^a^	F_2,4_ = 6.96	0.05
% Chitin (db) ^3^	17.95 ± 1.89 ^a^	24.57 ± 1.90 ^a^	24.94 ± 2.64 ^a^	F_2,3_ = 6.59	0.08
Astaxanthin (µg/g) (db) ^4^	58.45 ± 0.38 ^a^	59.32 ± 3.25 ^a^	60.78 ± 2.36 ^a^	F_2,6_ = 1.12	0.38

^1^ Results represent the mean of three determinations (*n* = 3) ± standard deviation.^2^ Results represent the mean of at least two determinations (*n* = 2) ± standard deviation and are reported on a dry weight basis (db). ^3^ Results represent the mean of 2 determinations (*n* = 2) ± standard deviation. Yield is reported as [mass chitin ÷ mass sample] × 100 and is reported on a dry weight basis (db). ^4^ Results represent the mean of three determinations (*n* = 3) ± standard deviation and are reported on a dry weight basis (db). ^5^ Values in the same row with different letters are significantly different (*p* < 0.05) according to Tukey’s test.

**Table 2:** Astaxanthin recovery from snow crab processing discards using vegetable oils ^1^.

% Astaxanthin in Vegetable Oil Solvents		
Sunflower Oil	Corn Oil	Canola Oil
24.85 ± 0.01 ^a^	37.93 ± 0.04 ^b^	31.23 ± 0.03 ^c^

^1^ Results are the mean of three replicates ± standard deviation. Total astaxanthin in crab processing by-products was 21.09 μg/g on a wet weight basis. Values with different letters are significantly different (F_2,6_ = 14.95, *p* = 0.0047) according to Tukey’s test.

**Table 3:** Composition of protein-pigment powders following carotenoid extraction with vegetable oils and demineralization with citric acid.

Composition	Sunflower Oil ^3^	Corn Oil ^3^	Canola Oil ^3^
% Protein ^1^ (db)	51.69 ± 0.53 ^a^	51.53 ± 1.23 ^a^	51.05 ± 0.52 ^a^
% Lipid ^2^ (db)	24.58	19.72	16.08
% Ash ^2^ (db)	0.86	0.72	0.76
Total Astaxanthin ^1^ (µg/g) (db)	39.56 ± 1.04 ^a^	33.77 ± 0.35 ^b^	37.67 ± 0.64 ^c^

^1^ Results are the mean of two replicates (*n* = 2) ± standard deviation and are reported on a dry weight basis (db). ^2^ Results represent one determination due to limited sample size available. Reported on a dry weight basis (db). ^3^ Values in the same row with different letters are significantly different (*p* < 0.05) according to Tukey’s test.

**Table 4:** Characteristics of chitins prepared from crab processing discards using a green chemistry approach.

Parameter	Sunflower Oil			Corn Oil			Canola Oil		
	FAP	Sea-B-Zyme	Weifang	FAP	Sea-B-Zyme	Weifang	FAP	Sea-B-Zyme	Weifang
% Chitin Yield ^a^ (db)	17.06	17.09	18.57	18.02	18.20	20.08	17.53	17.46	19.15
% Moisture ^b^	2.24 ± 0.68	1.65 ± 0.75	1.46 ± 0.50	1.10 ± 0.65	1.14 ± 0.37	1.26 ± 0.42	1.05 ± 0.55	1.26 ± 0.55	1.42 ± 0.42
% Ash ^c^ (db)	2.16	1.8	1.05	1.17	1.24	1.44	0.75	1.06	1.92
% Total Nitrogen ^b^ (db)	6.31 ± 0.10	6.34 ± 0.01	6.35 ± 0.03	6.38 ± 0.15	6.19 ± 0.01	6.61 ± 0.12	6.43 ± 0.07	6.24 ± 0.001	6.55 ± 0.21
% Residual protein ^d^ (db)	0.26 ± 0.17	0.39 ± 0.14	0.63 ± 0.21	0.28 ± 0.22	0.77 ± 0.37	0.95 ± 0.15	0.45 ± 0.28	0.76 ± 0.46	0.92 ± 0.15
% Chitin Nitrogen ^e^ (db)	6.27	6.28	6.25	6.34	6.07	6.46	6.30	6.12	6.40
Astaxanthin ^f^ (μg/g) (db)	17.61 ± 0.09	18.59 ± 0.57	9.91 ± 0.14	14.76 ± 0.77	20.70 ± 0.28	13.12 ± 0.10	15.64 ± 0.12	20.44 ± 0.21	12.36 ± 0.16
Hunter Color Parameters ^g^	FAP	Sea-B-Zyme	Weifang	FAP	Sea-B-Zyme	Weifang	FAP	Sea-B-Zyme	Weifang
L	65.76 ± 0.54	70.25 ± 0.51	67.08 ± 0.15	69.87 ± 0.26	68.89 ± 0.34	70.82 ± 0.47	66.35 ± 1.45	63.45 ± 1.06	66.76 ± 0.58
a	23.64 ± 0.11	23.58 ± 0.13	23.46 ± 0.04	23.48 ± 0.12	23.85 ± 0.10	22.45 ± 0.14	24.28 ± 0.37	24.35 ± 0.26	23.12 ± 0.23
b	3.44 ± 0.23	0.36 ± 0.79	1.28 ± 0.27	0.84 ± 0.31	0.26 ± 0.15	1.09 ± 0.29	−0.71 ± 0.39	−0.66 ± 0.64	1.25 ± 0.71

^a^ Results represent one determination based on extraction from ~ 100 g of raw crab by-product material. Reported on a dry weight basis (db). ^b^ Results represent the mean of two determinations (*n* = 2) ± standard deviation. ^c^ Results represent one determination due to the limited sample size available. Reported on a dry weight basis. ^d^ Results represent the mean of four determinations (*n* = 4) ± standard deviation. ^e^ Result calculated as the difference between total Kjeldahl nitrogen and Lowry protein nitrogen. ^f^ Results represent the mean of three determinations (*n* = 3) ± standard deviation. ^g^ Results represent the mean of six determinations (*n* = 6) ± standard deviation.

**Table 5:** Characteristics of chitins prepared from crab processing discards using a traditional chemical method.

				ANOVA	
Composition	May 2018 ^3^	June 2018 ^3^	July 2018 ^3^	F_2,3_	*p*-Value
% Chitin Yield ^1^ (db)	17.93 ± 1.89 ^a^	16.16 ± 5.65 ^a^	16.81 ± 0.31 ^a^	0.135	0.88
% Ash ^1^ (db)	0.68 ± 0.64 ^a^	0.46 ± 0.25 ^a^	0.26 ± 0.24 ^a^	0.48	0.66
% Total Nitrogen ^1^ (db)	6.30 ± 0.064 ^a^	6.38 ± 0.04 ^a^	6.43 ± 0.03 ^a^	4.53	0.12
% Residual protein ^1^ (db)	0.34 ± 0.36 ^a^	0.23 ± 0.25 ^a^	0.12 ± 0.02 ^a^	0.37	0.72
% Chitin Nitrogen ^1,2^ (db)	6.23 ± 0.014 ^a^	6.33 ± 0.014 ^a,b^	6.43 ± 0.049 ^b^	20.02	0.018

^1^ Results represent the mean of two determinations (*n* = 2) ± standard deviation. ^2^ Result calculated as the difference between total Kjeldahl nitrogen and Lowry protein nitrogen. ^3^ Values in the same row with different letters are significantly different (*p* < 0.05) according to Tukey’s test.

**Table 6:** Crystallinity index (CI) of snow crab chitin samples prepared in 2018 and 2021 ^1^.

Sample Description	I_am_ ^2^	I_110_ ^3^	% CI
Crab Shell Backs 2021 (Chemical Process)	500	3291.7	84.81
May 2021 (Chemical Process)	527.78	3097.2	82.96
May 2021 (Green Process: Corn Oil + FAP + H_2_O_2_)	611.11	3083.33	80.18
May 2018 (Chemical Process)	541.67	3000	81.94
June 2018 (Chemical Process)	333.33	4888.89	93.18
July 2018 (Chemical Process)	555.56	3555.56	84.37

^1^ Results represent one XRD scan (*n* = 1) per chitin sample. ^2^ Amorphous diffraction at 2θ. ^3^ Maximum intensity of the (110) peak at around 2θ.

**Table 7:** Enzyme characteristics and reaction parameters for enzymatic deproteination of demineralized snow crab shells.

Parameter	Fungal Acid Protease (*Aspergillus oryzae)*	Sea-B-Zyme L200	Weifang Acid Protease
Type of Protease	Endo and Exo	Acid Protease	Acid Protease
Enzyme Activity	>500 U/g	Not specified	>100,000 U/g
% Enzyme ^1^	2%	2%	2%
pH	7.0	5.0	3.0
Optimum Temp Range	30–55 °C	40–55 °C	45 °C
Reaction Temp	45 °C	45 °C	45 °C
Shell:Water	1:10	1:10	1:10
Bulk Cost (USD/kg) ^2^	80.00	32.50	12.00

^1^ Enzyme concentration = % (*w*/*w*) of demineralized shell. ^2^ 2021 prices.

**Table 8:** Methods used for characterization of snow crab bio-products.

Crab Bio-Product	Parameters Analyzed	Method
Pigmented Oils	Total Astaxanthin	hexane:isopropanol [41], Equation (4)
Protein-Pigment Powder	Moisture	AOAC Method 930.14
	Total Nitrogen	AOAC Method 954.01/988.05
	Ash Content	AOAC Method 938.08 Ash of Seafood
	Total Astaxanthin	hexane:isopropanol [41], Equation (4)
Chitin	Moisture	AOAC Method 930.14
	Total Nitrogen	AOAC Method 954.01/988.05
	Protein Nitrogen	Lowry method, Equation (3)
	Ash Content	AOAC Method 938.08 Ash of Seafood
	Total Astaxanthin	hexane:isopropanol [41], Equation (4)
	Chitin Yield	Equation (6)
	Chitin Nitrogen	Equation (2)
	Tristimulus Color Parameters	ColorTec PCM Colorimeter
	Powder X-ray Diffraction	Rigaku Ultima IV X-ray diffractometer, Equation (5)

## Data Availability

The data presented in this study are available in this article in Table 1, Table 2, Table 3, Table 4 and Table 5. Correlation coefficients for astaxanthin and L*a*b* values of chitin are available in Appendix A, Table A1.

## Appendix A

**Table A1:** Correlation coefficients, r, for astaxanthin and L*a*b* values of chitin ^1,2^.

	AXT	*L*	*a*	*b*
AXT	1			
*L*	−0.19	1		
*A*	0.60	−0.64	1	
*B*	−0.29	0.06	−0.48	1

^1^ AXT = astaxanthin, *L* = L-value (whiteness), *a* = a-value (redness), *b* = b-value (blueness).^2^ Correlations were not significant (*p* > 0.05).
